# Preliminary study of anti-CD40 and ubiquitin proteasome antibodies in primary podocytopaties

**DOI:** 10.3389/fmed.2023.1189017

**Published:** 2023-06-20

**Authors:** Natalia Chebotareva, Venzsin Cao, Anatoliy Vinogradov, Igor Alentov, Natalia Sergeeva, Alexey Kononikhin, Sergey Moiseev

**Affiliations:** ^1^Sechenov First Moscow State Medical University, Tareev Clinic of Internal Diseases, Moscow, Russia; ^2^Lomonosov Moscow State University, Faculty of Medicine, Moscow, Russia; ^3^Hertsen Moscow Oncology Research Institute, Department of Prediction of Conservative Treatment Efficiency, Moscow, Russia; ^4^Skolkovo Institute of Science and Technology, Moscow, Russia

**Keywords:** podocytopathy, FSGS, anti-CD40 antibodies, anti-UCH-L1 antibodies, minimal change disease

## Abstract

**Background:**

Minimal change disease and focal segmental glomerulosclerosis are primary podocytopathies that are clinically presented in adults presenting with severe nephrotic syndrome. The pathogenesis of these diseases is not clear and many questions remain to be answered. A new concept about the role of changes in the antigenic determinant of podocytes and the production of anti-podocyte antibodies that cause podocyte damage is being developed. The aim of the study is to evaluate the levels of anti-CD40 and anti-ubiquitin carboxyl-terminal hydrolase L1 (anti-UCH-L1) antibodies in patients with podocytopathies in comparison with other glomerulopathies.

**Methods:**

One hundred and six patients with glomerulopathy and 11 healthy subjects took part in the study. A histological study revealed primary FSGS in 35 patients (genetic cases of FSGS and secondary FSGS in the absence of NS were excluded), 15 had MCD, 21 - MN, 13 - MPGN, 22 patients - IgA nephropathy. The effect of steroid therapy was evaluated in patients with podocytopathies (FSGS and MCD). The serum levels of anti-UCH-L1 and anti-CD40 antibodies were measured by ELISA before steroid treatment.

**Results:**

The levels of anti-UCH-L1 antibodies were significantly higher in MCD patients and anti-CD40 antibodies were higher in MCD and FSGS than in the control group and other groups of glomerulopathies. In addition, the level of anti-UCH-L1 antibodies was higher in patients with steroid-sensitive FSGS and MCD, and anti-CD40 antibodies were lower than in patients with steroid-resistant FSGS. An increase in anti-UCH-L1 antibody levels above 6.44 ng/mL may be a prognostic factor of steroid-sensitivity. The ROC curve (AUC = 0.875 [95% CI 0.718–0.999]) for response to therapy showed a sensitivity of 75% and specificity of 87.5%.

**Conclusion:**

An increase in the level of anti-UCH-L1 antibodies is specific for steroid-sensitive FSGS and MCD, while an increase in anti-CD40 antibodies – for steroid-resistant FSGS, compared with other glomerulopathies. It suggests that these antibodies could be a potential factor for differential diagnosis and treatment prognosis.

## Introduction

### Study subjects

Primary podocytopathies, focal segmental glomerulosclerosis (FSGS) and minimal change disease (MCD), are a common group of glomerular disorders that lead to a high proteinuria and nephrotic syndrome (NS). MCD often develops in children; it is characterized by its good response to steroid therapy, but at the same time by the frequent development of steroid-dependent and often relapsing NS. FSGS is one of the main causes of steroid-resistant NS and progresses to terminal-stage chronic kidney disease in about a third of all cases. The incidence rate of FSGS is about 15–35% of histologically confirmed glomerulopathies with proteinuria and NS (1, 2). Through kidney biopsies it has been noted that the frequency of FSGS has increased by 41% over the past 10 years, approaching diabetic nephropathy (3, 4).

The pathogenesis of primary FSGS and MCD is currently unknown. It is assumed that progressive damage to podocytes in primary FSGS and MCD is associated with circulating permeability factors in the blood of these patients. Savin VJ and Sharma M isolated a certain circulating factor with a molecular weight of approximately 50 kDa using the chromatographic method. However, this «factor» has not been identified yet (5–7). The most likely candidates are cytokines, as well as cell receptors and growth factors - urokinase plasminogen activator soluble receptor type (suPAR) and many other factors isolated from the serum of patients with recurrent FSGS (5, 8–14). Some studies have demonstrated the role of auto-antibodies in idiopathic nephrotic syndrome in children (15, 16) as well as in adults with MCD (17). Recently, some research groups have suggested the role of antibodies such as anti-ubiquitin-C-terminal hydrolase L1 antibodies (anti-UCH-L1 antibodies) and anti-CD40 antibodies in podocytopathies (18, 19). In the serum of patients with recurrent FSGS, an increase in the levels of these antibodies indicates a possible involvement of the B cell in the pathogenesis of podocytopathies (17–19). However, further evaluation is required in order to determine their significance as specific factors of podocyte.

The aim of our study was to assess the level of anti-UCH-L1 antibodies and anti-CD40 antibodies as potential factors of podocyte damage in patients with different glomerulopathies, and to evaluate their significance in diagnosis of primary podocyte diseases.

## Materials and methods

The study included 106 patients with glomerulopathies, 46 women (43.4%) and 60 men (56.6%) aged 18–71 years (median 37.0 [29.7–50] years). The control group included 11 healthy individuals: 5 (45%) women and 6 (55%) men aged 23–60 (median 34 [30–48] years).

The study was approved by the Ethics Committee of Sechenov University. Conforming with the Declaration of Helsinki, all subjects provided their informed written consents before participating in the study.

The severity of tubulointerstitial fibrosis was assessed using a semi-quantitative score: + <25%, ++ 25–50%, and +++ interstitial fibrosis >50% (20). The number of sclerosed glomeruli was assessed as the percentage of sclerosed glomeruli from the total number of glomeruli in the biopsy.

The response to steroid therapy and the time to achieve remission were evaluated in patients with podocytopathies (MCD and FSGS). A decrease in proteinuria to less than 300 mg/day with a stable renal function refered to complete remission; a decrease in proteinuria by 50% or more from baseline values with normalization of serum proteins was defined as partial remission (21). The failure to respond to steroids for more than 16 weeks with satisfactory tolerance and more than 8 weeks with poor tolerance was considered a no response to steroids.

### Determination of anti-UCH-L1 and anti-CD40 antibodies in serum

In order to detect antibodies, a serum was obtained by the centrifugation of the whole blood at room temperature for 20 min at 3,000 rpm. The antibodies to ubiquitin-C-terminal hydrolase L1 (anti-UCH-L1 antibodies) (Cloud-Clone Corp., BlueGene, Elabscience Biotechnology, США), and anti-CD40 antibodies (Cloud-Clone Corp., BlueGene, Elabscience Biotechnology, США) were studied using enzyme immunoassay.

The results were analysed using the methods of variation statistics from the SPSS 23 and Jamovi software packages.

## Results

Histological examination revealed primary FSGS in 35 patients, MCD in 15, MN in 21, MPGN in 13 (cases of immune-complex MPGN that were not associated with cryoglobulinemia, infections, including hepatitis C and B viruses, systemic diseases, monoclonal gammopathy), and IgA nephropathy in 22 patients. Sixty-five patients (60.7%) had NS at the onset, impaired renal function (eGFR according to the CKD-EPI formula less than 60 mL/min/1.73 m^2^ (22)) was observed in 46 (43%) patients. The study included cases of FSGS with acute onset and with development of NS (proteinuria more than 3.5 g/day, hypoalbuminemia less than 30 g/L, hyperlipidemia). Patients with no NS (secondary FSGS) at onset and patients with a family history of kidney disease were not included in the study. The median duration of therapy until response in steroid-sensitive cases was 1 [0.26; 5.13] month; in the group with steroid-resistant cases the total follow-up period was 31 [8.8; 58.5] months. The characteristics of the patients examined are presented in Table 1.

**Table 1:** Characteristics of the patients examined.

Parameters	FSGS *n* = 35	MCD *n* = 15	MN *n* = 21	MPGN *n* = 13	IgA nephropathy *n* = 22
Age, years	35 [19–69]	30 [18–59]	46 [26–71]	38 [22–67]	34 [19–67]
Males, *n* (%)	19 (48.7)	5 (33.3)	17 (81)	7 (63.6)	12 (57.14)
Proteinuria, g/24 h	3.8 [2.03–15.5]	5.7 [3.93–9.92]	3.6 [1.11–7.9]	3.7 [0.8–8.15]	2.7 [0.1–9]
Serum albumin, g/l	22.5 [20.0–28.0]	20.8 [12.9–24.9]	29.6 [19.8–36.7]	32.4 [19.6–45.4]	35.7 [20.2–47]
Nephrotic syndrome, *n* (%)	35 (100)	15 (100)	16 (76)	8 (61.5)	6 (27)
Serum creatinine, μmol/l	108.0 [46–198]	76.3 [50–144.8]	89 [64–146.3]	130,3 [75–200]	135 [56.9–200]
eGFR, ml/min/1,73 m^2^	62.2 [24–127]	91 [40–140]	78.8 [41–118]	51 [23–101]	54 [33–132.9]
Arterial hypertension, *n* (%)	26 (66.7)	5 (33.3)	12 (57.14)	10 (90.9)	17 (81)
Steroid-resistant NS, *n* (%)	16 (45.7%)	0			
Steroid-sensitive NS, *n* (%)	19 (54.3%)	15 (100%)			
Complete remission, *n* (%)	10 + 15				
Partial remission, *n* (%)	9 (20.7%)	0			

The table shows the median [min; max].

### The level of antibodies to ubiquitin-C-terminal hydrolase L1 and anti-CD40 antibodies in serum of patients with glomerulopathies

**Figure 1:**
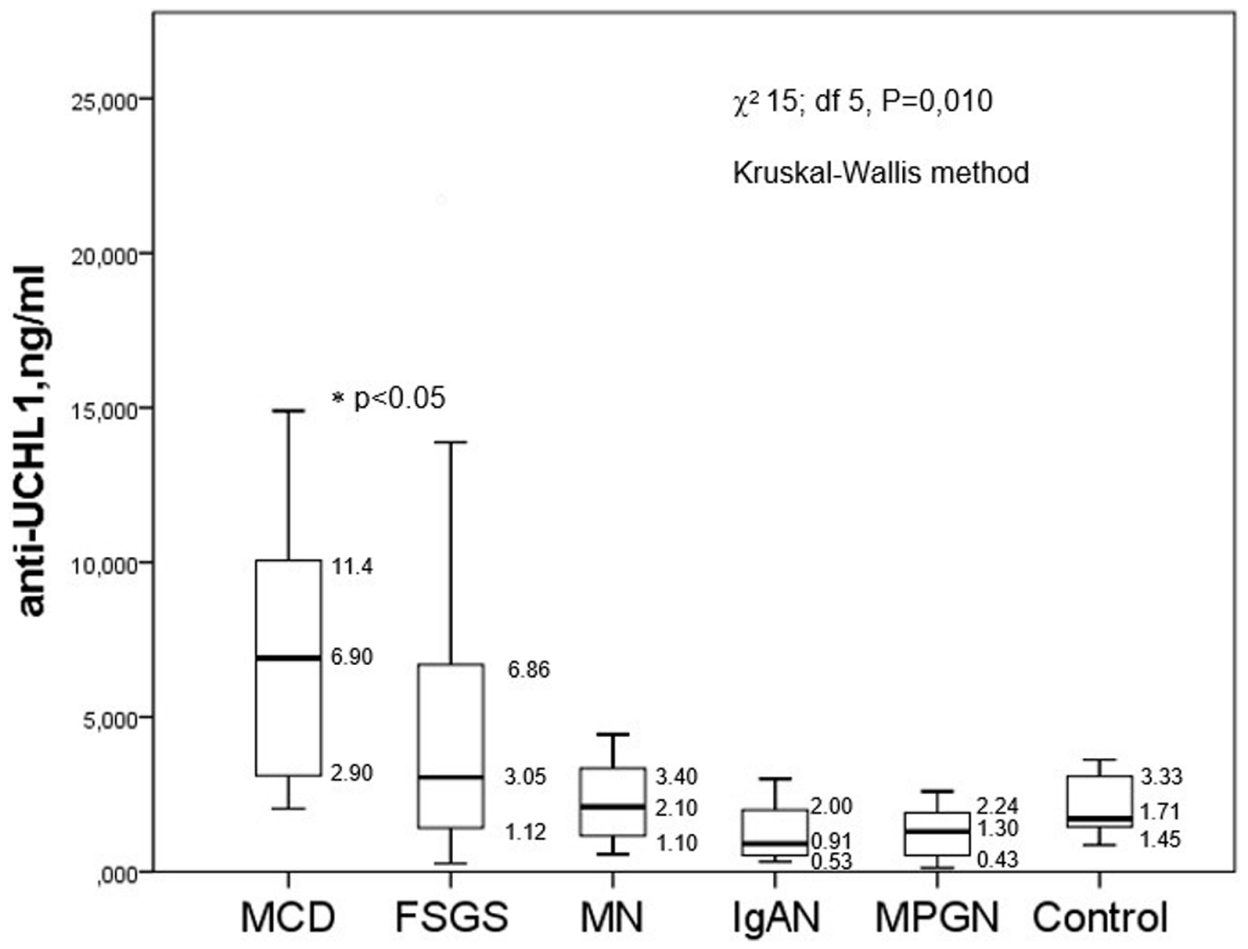
The baseline levels of anti-UCH-L1 antibodies in serum of patients with different glomerulopathies.

The level of anti-UCH-L1 antibodies was significantly higher in patients with MCD than in the control group and in other glomerulopathies - FSGS, membranous nephropathy, IgA nephropathy and membranoproliferative glomerulonephritis. However, the difference between the minimum and maximum levels of anti-UCH-L1 antibodies was very high in FSGS patients (Figure 1).

There were no significant correlations between the level of anti-UCH-L1 antibodies in the blood serum and daily proteinuria (Rs = 0.073, *p* = 0.454), serum albumin (Rs = −0.196, *p* = 0.160), the percentage of sclerotic glomeruli (Rs = −0.102, *p* = 0.425) or the tubulointerstitial fibrosis score (Rs = −0.187, *p* = 0.142), however, there was a weak correlation between anti-UCH-L1 antibodies serum and creatinine/GFRCKD-EPI (Rs = −0.301, *p* = 0.002/Rs = 0.318, *p* = 0.007, respectively).

**Figure 2:**
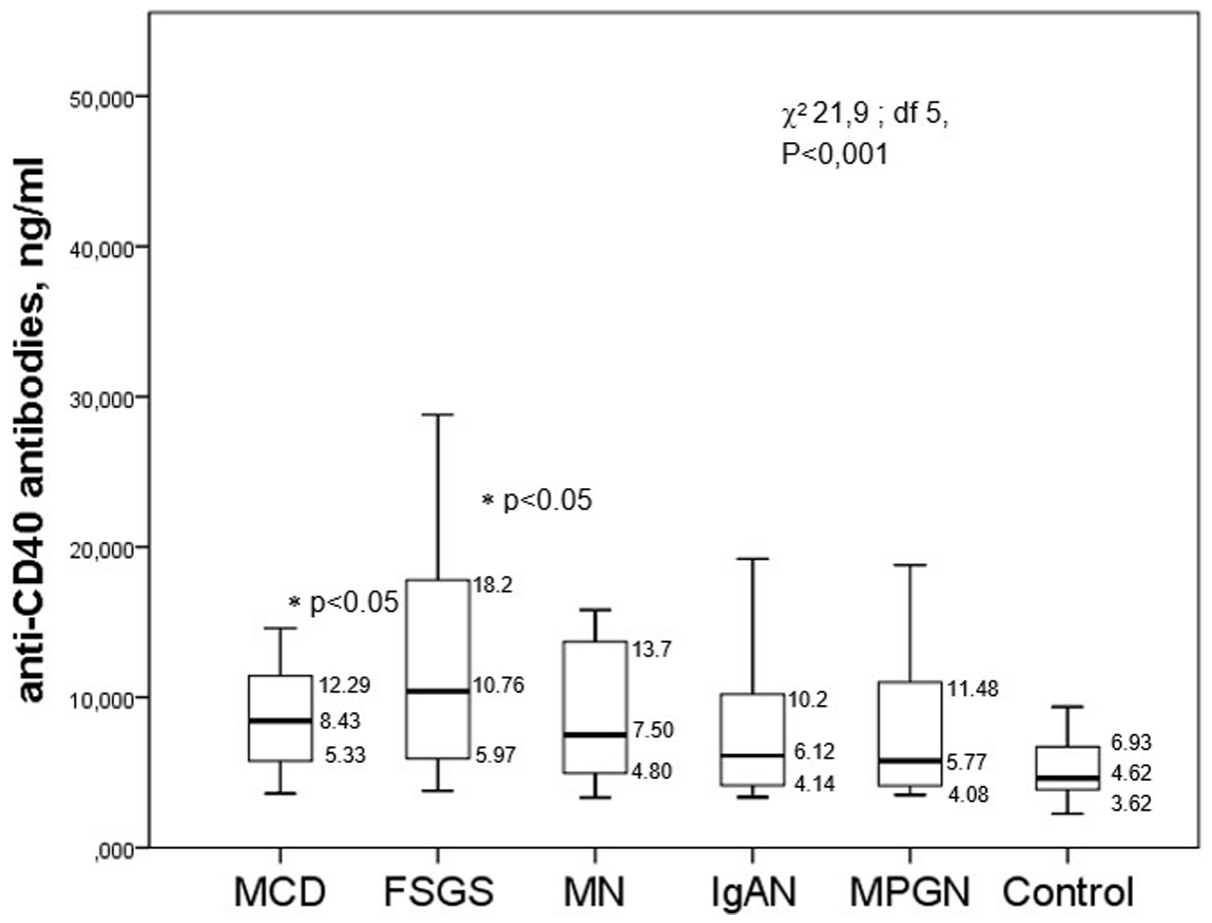
The level of anti-CD40 antibodies in the serum of patients with different glomerulopathies.

The median serum levels of anti-CD40 antibodies were significantly higher in the FSGS and MCD groups than in the controls and other glomerulopathies (membranous nephropathy, IgA nephropathy, and MPGN). However, there was also a difference in the levels of antibodies in the FSGS group as compared to other glomerulopathies (Figure 2).

There were no significant correlations between the level of anti-CD40 antibodies with the creatinine serum/eGFR_CKD-EPI_ (Rs = −0.057, *p* = 0.561 and Rs = 0.124, *p* = 0.283, respectively), the percentage of sclerotic glomeruli (Rs = −0.051, *p* = 0.679) or the tubulointerstitial fibrosis score (Rs = 0.048, *p* = 0.7), though there was a likely correlation with daily proteinuria (Rs = 0.153, *p* = 0.11) and a negative correlation with the albumin serum (Rs = −0.275, *p* = 0.016).

### Response to corticosteroid therapy depending on the baseline levels of anti-UCH-L1 and anti-CD40 antibodies

**Figure 3:**
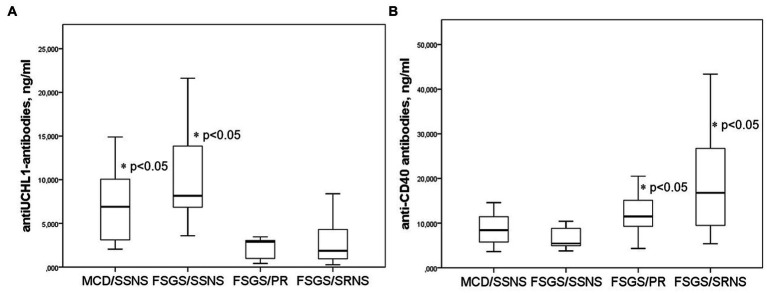
Anti-UCH-L1 antibodies **(A)** and anti-CD40 antibodies **(B)** in patients with different response to corticosteroid therapy. SSNS, steroid-sensitive NS; SRNS, steroid-resistant NS; PR, partial remission.

The baseline level of anti-UCH-L1 antibodies (before corticosteroid administration) were significantly higher in steroid-sensitive than in steroid-resistant cases. At the same time, the baseline levels of anti-UCH-L1 antibodies were higher in the group of patients with steroid-sensitive NS (MCD and FSGS) who achieved complete remission; lower antibody levels were found in FSGS patients with partial remission or no response to corticosteroids (Figure 3A).

In contrast, the baseline level of anti-CD40 antibodies were higher in patients with severe steroid-resistant NS compared to those who responded to steroid therapy. The level of anti-CD40 antibodies was lower in steroid-sensitive NS, and higher in patients with partial remission or no response to corticosteroids (Figure 3B).

ROC analysis was applied to assess the sensitivity and specificity of the use of antibodies for the prognosis of steroid response during 1 month of therapy in patients with MCD and FSGS. Complete response to steroid therapy can be predicted at a level of anti-UCH-L1 antibodies above 6.44 ng/mL (sensitivity 75%, specificity 87.5%), AUC 0.875 [0.718–0.999], *p* < 0.023 and with the level of anti-CD40 antibodies below 9.65 ng/mL AUC = 0.698 [0.515–0.881], *p* < 0.045 (sensitivity 80%, specificity 71.4%).

## Discussion

Simultaneous with the hypothesis of T-cell dysfunction underlying podocytopathies – MCD and FSGS, there is another point of view that the development of these diseases can be associated with the activation of B cells, and podocyte-specific antibodies can cause damage to podocytes. For example, annexin A2 autoantibody was detected in 17.8% of children with MCD or FSGS, anti-actin and ATP- synthase – in 16.7% of children with idiopathic nephrotic syndrome (15, 16). These studies only included children with idiopathic nephrotic syndrome. Studies in adults have not yet been published. However, circulating nephrin autoantibodies were detected in almost one-third of the patients, both children and adults enrolled in the Nephrotic Syndrome Study Network (NEPTUNE) cohort with active proteinuria and this supports the autoimmune etiology in a subset of MCD patients confirming similar mechanisms of this disease in children and adults (17).

The involvement of antibodies in the pathogenesis is consistent with the complete reversibility of NS in steroid-sensitive cases due to the rapid recovery of podocytes. Rituximab causes B-cell depletion and can also induce remission in patients with a steroid-dependent and relapsing form of the disease. According to some studies, an increase in the number of memory B cells in MCD/FSGS might be a predictor of relapse after discontinuation of therapy (23, 24). It cannot be excluded that several different antibodies are involved in the pathogenesis of MCD and FSGS, which can determine the course of the disease and the response to therapy.

Jamin et al. were the first to show the role of anti-UCH-L1 IgG antibodies in patients with recurrent idiopathic nephrotic syndrome. These human-isolated antibodies were able to induce proteinuria and the effacement of podocyte processes in mice (19). In a study by Jamin et al. an increase in anti-UCH-L1 antibodies levels was observed only in children with INS, while in adults with MCD and NS it did not differ from the control group (19). However, the number of patients was quite small. In addition, patients who had an early stage FSGS or genetic variants could be included in this group. To test the hypothesis about the role of anti-UCH-L1 antibodies in podocytopathy in adults, we studied the levels of anti-UCH-L1 antibodies in the serum of patients with different glomerular diseases. Higher levels of anti-UCH-L1 antibodies have been detected in patients with MCD compared with the control groups, patients with MN, IgA-nephropathy, MPGN, and FSGS. However, when dividing FSGS into groups of steroid-sensitive and steroid-resistant NS, it is important to highlight that anti-UCH-L1 antibodies levels are higher in patients with steroid-sensitive NS regardless of MCD or FSGS and this may predict the response to steroid therapy.

Although the mechanism of damage by anti-UCH-L1 antibodies is still not known, it is hypothesized that antibodies may induce podocyte apoptosis either through direct action or indirectly through antibody-dependent cellular cytotoxicity. Another hypothesis is that anti-UCH-L1 antibodies can undergo endocytosis or transcytosis through podocytes *via* the neonatal Fc receptor (25). An increase in the intracellular concentration of the ubiquitin hydrolase can lead to protein degradation due to the deubiquitinating processes in podocytes (26). This allows us to discuss the important role of the ubiquitin system in maintaining the podocyte cytoskeleton and its possible damage by anti-UCH-L1 antibodies. The association of high antibodies levels with retained renal function and the rapid response to corticosteroids reflects an early stage of podocytopathy and the reversibility of damage.

We noted an increase in the level of anti-CD40 antibodies in the serum of patients with FSGS and MCD compared with other forms of glomerulopathies. In addition, the results of our study support this idea, as high levels of anti-CD40 antibodies are associated with severe steroid resistant FSGS. The key question is the reason for the production of anti-CD40 antibodies. The cell-surface molecule CD40, a member of the tumor necrosis factor receptor superfamily, broadly regulates immune activation and mediates tumor apoptosis. Agonistic CD40 antibodies have been shown to substitute for T cell help provided by CD4+ lymphocytes in murine models of T cell-mediated immunity (27). CD40 can manifest itself on damaged podocytes (18). Anti-podocyte antibodies may be the result of a secondary response to the expression of neoantigens on the surface of the podocyte which could also be the reason why anti-antibodies do not damage other cell types. For example, the neo-expression of CD40 on the surface of podocytes has been detected in patients with FSGS recurrence after kidney transplantation (18). Possibly, the main localization of podocyte damage in FSGS is the area of interaction of podocyte integrins with the actin cytoskeleton of podocytes. While suPAR can bind the αvβ3 integrin on the surface of podocytes, promoting podocyte detachment from the GBM (28, 29), anti-CD40 antibodies seem to prolong the suPAR-mediated activation of integrins and/or the corresponding signaling pathways, resulting in increased podocyte motility and their massive loss *in vivo* (18). It is possible that primary suPAR-mediated damage to podocytes leads to the unmasking of some podocyte epitopes - CD40, and an antibody response is formed. A combination of abnormalities in certain antigen epitopes may also explain the increased autoantibodies levels observed in FSGS patients. Autoantibodies targeting podocyte proteins can attack glomeruli and cause damage of the glomerular filtration barrier (30).

High levels of antibodies to ubiquitin hydrolase and lower levels of anti-CD40 antibodies can predict the response to steroids during 1 month of treatment with high doses of corticosteroids.

Our study has limitations. The number of patients was relatively small, especially patients with MCD, which is one of the most common causes of nephrotic syndrome in children but not in adults. We did not study СD40 expression in renal tissue. We did not check how the level of antibodies changes during the treatment of patients or the progression of renal dysfunction. To consider these antibodies as podocytopathy markers, further study is required.

## Conclusion

Anti-СD40 and anti-ubiquitin carboxyl-terminal hydrolase L1 antibodies increase in primary podocytopaties. Based on the level of antibodies, patients with MCD and FSGS can be divided into two subgroups: patients with steroid-sensitive MCD and FSGS are characterized by high level of anti-UCH-L1 antibodies and a low levels of anti-CD40 antibodies; while patients with steroid-resistant FSGS, on the contrary, are characterized by high levels of anti-CD40 antibodies and low levels of anti-UCH-L1 antibodies. The data may reflect on the different pathogenetic mechanisms of a sensitive and resistant form of FSGS.

## Data availability statement

The raw data supporting the conclusions of this article will be made available by the authors, without undue reservation.

## Ethics statement

The studies involving human participants were reviewed and approved by the Ethics Committee of the Sechenov University. The patients/participants provided their written informed consent to participate in this study.

## Author contributions

NC: conceptualization. NC, AV, and AK: methodology. NC, VC, IA, and AV: investigation. NC, AV, and NS: writing – original draft preparation. SM and AK: writing – review and editing. All authors contributed to the article and approved the submitted version.

## Funding

This research was funded by the Russian Science Foundation, grant #21-74-20173.

## Conflict of interest

The authors declare that the research was conducted in the absence of any commercial or financial relationships that could be construed as a potential conflict of interest.

## Publisher’s note

All claims expressed in this article are solely those of the authors and do not necessarily represent those of their affiliated organizations, or those of the publisher, the editors and the reviewers. Any product that may be evaluated in this article, or claim that may be made by its manufacturer, is not guaranteed or endorsed by the publisher.
